# Hypothesis: The Psychedelic Ayahuasca Heals Traumatic Memories via a Sigma 1 Receptor-Mediated Epigenetic-Mnemonic Process

**DOI:** 10.3389/fphar.2018.00330

**Published:** 2018-04-05

**Authors:** Antonio Inserra

**Affiliations:** ^1^Mind and Brain Theme, The South Australian Health and Medical Research Institute, Adelaide, SA, Australia; ^2^Department of Psychiatry, College of Medicine and Public Health, Flinders University, Adelaide, SA, Australia; ^3^Centre for Neuroscience, Flinders University, Adelaide, SA, Australia

**Keywords:** Ayahuasca, DMT, sigma 1 receptor, trauma, post-traumatic stress disorder, epigenetics, fear extinction, cellular memory

## Abstract

Ayahuasca ingestion modulates brain activity, neurotransmission, gene expression and epigenetic regulation. *N,N*-Dimethyltryptamine (DMT, one of the alkaloids in Ayahuasca) activates sigma 1 receptor (SIGMAR1) and others. SIGMAR1 is a multi-faceted stress-responsive receptor which promotes cell survival, neuroprotection, neuroplasticity, and neuroimmunomodulation. Simultaneously, monoamine oxidase inhibitors (MAOIs) also present in Ayahuasca prevent the degradation of DMT. One peculiarity of SIGMAR1 activation and MAOI activity is the reversal of mnemonic deficits in pre-clinical models. Since traumatic memories in post-traumatic stress disorder (PTSD) are often characterised by “repression” and PTSD patients ingesting Ayahuasca report the retrieval of such memories, it cannot be excluded that DMT-mediated SIGMAR1 activation and the concomitant MAOIs effects during Ayahuasca ingestion might mediate such “anti-amnesic” process. Here I hypothesise that Ayahuasca, via hyperactivation of trauma and emotional memory-related centres, and via its concomitant SIGMAR1- and MAOIs- induced anti-amnesic effects, facilitates the retrieval of traumatic memories, in turn making them labile (destabilised). As Ayahuasca alkaloids enhance synaptic plasticity, increase neurogenesis and boost dopaminergic neurotransmission, and those processes are involved in memory reconsolidation and fear extinction, the fear response triggered by the memory can be reprogramed and/or extinguished. Subsequently, the memory is stored with this updated significance. To date, it is unclear if new memories replace, co-exist with or bypass old ones. Although the mechanisms involved in memory are still debated, they seem to require the involvement of cellular and molecular events, such as reorganisation of homo and heteroreceptor complexes at the synapse, synaptic plasticity, and epigenetic re-modulation of gene expression. Since SIGMAR1 mobilises synaptic receptor, boosts synaptic plasticity and modulates epigenetic processes, such effects might be involved in the reported healing of traumatic memories in PTSD patients. If this theory proves to be true, Ayahuasca could come to represent the only standing pharmacological treatment which targets traumatic memories in PTSD. Lastly, since SIGMAR1 activation triggers both epigenetic and immunomodulatory programmes, the mechanism here presented could help understanding and treating other conditions in which the cellular memory is dysregulated, such as cancer, diabetes, autoimmune and neurodegenerative pathologies and substance addiction.

## Introduction

Ayahuasca is a psychoactive plant brew containing *N,N*-dimethyltryptamine (DMT) and β-carboline alkaloids (harmine, harmaline, and tetrahydroharmine) traditionally used in the Amazon basin for therapeutic and spiritual purposes (Schultes et al., 1979; Frecska et al., 2016). The hallucinogenic tryptamine DMT is obtained from *Psychotria viridis* and it binds to SIGMAR1, the serotonin receptors (5HTR) 1A/1D/1E/2A/2B/2C/5A/6/7, the serotonin transporter, the dopamine receptor D1 (D1R), the adrenergic receptors alpha 1A/1B/2A/2B/2C, the imidazoline 1 receptor and the trace amine associated receptor (Deliganis et al., 1991; Smith et al., 1998; Bunzow et al., 2001; Fontanilla et al., 2009; Ray, 2010). β-carbolines are obtained from *Banisteriopsis caapi* and function as monoamine oxidase inhibitors (MAOIs) to render DMT orally active (Riba et al., 2003).

Ayahuasca seems to hold therapeutic potential in psychiatry. Recently, fast onset antidepressant effects were reported following administration of a single dose of Ayahuasca in patients diagnosed with recurrent depression (Sanches et al., 2016). Similarly, anecdotal evidence suggests that Ayahuasca might be beneficial in the treatment of post-traumatic stress disorder (PTSD) (Nielson and Megler, 2014). However, no pre-clinical or clinical studies to date have investigated this possibility.

In this work, based on converging layers of evidence from *in-vitro*, pre-clinical and clinical studies, I postulate a mechanism involving the activation of discrete brain areas and receptor systems which triggers the recall of traumatic memories and their reconsolidation (and potentially fear extinction learning) hypothetically via modifying the epigenetic signatures of the memory.

## Ayahuasca Ingestion Modulates Brain Activity

The deep changes in perception and cognition elicited by Ayahuasca ingestion are underlined by a profound activation of limbic, paralimbic and neocortical brain areas, which are involved in trauma, memory formation, memory retrieval and emotional regulation, as well as a region-specific shift of electrical activity. These changes lead to an altered state of awareness underlined by introspection, retrieval of traumatic memories, and visions. Imaging studies have shown that Ayahuasca hyperactivates the inferior frontal gyrus (IFG) and the anterior insula, the right anterior cingulate/subcallosal gyrus and the left amygdala/parahippocampal gyrus, while decreasing activity within relevant hubs of the default mode network (DMN), such as the precuneus/posterior cingulate cortex and the medial prefrontal cortex (Riba et al., 2006; Palhano-Fontes et al., 2015).

The inferior frontal gyrus (IFG) is a brain area involved in semantic unification, emotion perception and regulation and processing of negative emotional stimuli (Etkin et al., 2011; Zhu et al., 2012; Tabei, 2015; Urgesi et al., 2016). This suggests that activation of this brain area during Ayahuasca ingestion could be involved in the processing of trauma. Significantly, veterans diagnosed with PTSD display decreased IFG activation in response to contextual cues, suggesting that modulation of this brain area might be beneficial in PTSD treatment (van Rooij et al., 2014). Similarly, the anterior insula is hyperactivated following Ayahuasca ingestion, and this region is involved in emotional processing and in the conscious perception of errors (Phillips et al., 1998; Ullsperger et al., 2010). The amygdala is involved in fear response, emotional arousal processes, reconsolidation of fear memories and fear memory extinction, and this brain region has been shown to be hyper-responsive in PTSD (Riba et al., 2006; Shin et al., 2006; Myers and Davis, 2007; Palhano-Fontes et al., 2015). Ayahuasca-induced hyperactivity of this brain area therefore supports the processing and reconsolidation of traumatic memories and the extinction of the fear memory associated with recall of the traumatic memory (Riba et al., 2006; Shin et al., 2006; Myers and Davis, 2007; Palhano-Fontes et al., 2015).

The subcallosal gyrus is involved in the processing of sadness and sad memories (Mayberg et al., 1999). Increased activity of this brain region is observed when patients are asked to rehearse sad autobiographic scripts, in line with the hypothesis presented here. Significantly, co-activation of the IFG, amygdala and hippocampus is a prerequisite for autobiographical memory retrieval, and specific sub-regions of these brain areas are activated following Ayahuasca ingestion (Greenberg et al., 2005; Riba et al., 2006). Moreover, parahippocampal gyrus activity is increased during memory retrieval tasks, and Ayahuasca hyperactivates this brain area (Maguire and Mummery, 1999).

Much like other psychedelic compounds such as psilocybin and lysergic acid diethylamide (LSD), Ayahuasca ingestion dampens activity and connectivity of crucial hubs within the DMN, such as the precuneus/posterior cingulate cortex and the medial prefrontal cortex (Carhart-Harris et al., 2012; Palhano-Fontes et al., 2015; Speth et al., 2016). Of relevance, the medial prefrontal cortex is involved in the process of fear extinction, and its activity is modulated by Ayahuasca (Myers and Davis, 2007; Palhano-Fontes et al., 2015).

## Ayahuasca Ingestion Modulates Neurotransmission

To date, only one study has investigated the effects of Ayahuasca administration on neurotransmission (de Castro-Neto et al., 2013). In this study, the authors orally administered rats three Ayahuasca doses and studied *post-mortem* amino acid and monoamines levels in the hippocampus and amygdala. Gamma-aminobutyric acid (GABA), the main inhibitory neurotransmitter in the human brain, was dose-independently increased in the hippocampus while it was increased in the amygdala at the lowest dose and decreased at the highest concentrations. Moreover, in the amygdala, noradrenaline, serotonin and dopamine levels were increased at all doses studied, while in the hippocampus only serotonin was increased in rats receiving the two highest doses. Furthermore, the turnover of serotonin, noradrenaline and dopamine was drastically reduced in the amygdala but not in the hippocampus of Ayahuasca-treated rats (de Castro-Neto et al., 2013).

These findings suggest that Ayahuasca ingestion exerts profound monoaminergic effects in the amygdala, increasing the levels of excitatory and decreasing those of inhibitory neurotransmitters, while decreasing monoamine utilisation. The findings that GABA is decreased and dopamine is increased in the amygdala following Ayahuasca administration is relevant for the hypothesis here presented, since the GABAergic system mediates the amnesic effects of chemical compounds, and negative modulation of the GABAergic system has anti-amnesic effects, while amygdalar dopamine is involved in the extinction of conditioned fear (Rau et al., 2009; Abraham et al., 2014). Thus, the decreased levels of amygdalar GABA might be at least partially responsible for the anti-amnesic-like effects of Ayahuasca on the retrieval of repressed memories in PTSD patients, while the increased levels of amygdalar dopamine might play an important role in the process of fear extinction (discussed below).

Further studies should investigate if similar changes in neurotransmission are replicable in humans. This could be possible via *in vivo* approaches, by using neuroimaging techniques to a) directly measure the levels of neurotransmitter release following Ayahuasca ingestion or b) indirectly, by measuring the relative drug occupancy at receptors for each of the neurotransmitter of interest (Badgaiyan, 2014; Kumar and Mann, 2014). However, until otherwise proven, it seems likely that Ayahuasca ingestion might trigger similar neurotransmission patterns in humans. These changes could be involved in the reported antidepressant effects of Ayahuasca and in the anecdotal reports of Ayahuasca consumption in the healing of trauma (Dominguez-Clave et al., 2016; Sanches et al., 2016).

## Ayahuasca Ingestion Modulates Neurogenesis

Aside from their monoaminergic effects, the alkaloids present in Ayahuasca have been shown to increase neurogenesis *in vitro* and *in vivo* at least partially via SIGMAR1-mediated upregulation of brain-derived neurotrophic factor (BDNF) (Fortunato et al., 2009; Fujimoto et al., 2012; Lenart et al., 2016; Morales-Garcia et al., 2017). The processes of memory reconsolidation and fear extinction (discussed below) both require synaptic plasticity enhancement and hippocampal neurogenesis, which are modulated by BDNF (Radiske et al., 2015; Suarez-Pereira and Carrion, 2015). Therefore, it seems likely that the Ayahuasca-induced synaptic plasticity and neurogenesis, which are required for mnemonic processes and fear extinction, might be involved in the healing of traumatic memories experienced by Ayahuasca users.

## Sigma-1 Receptor

SIGMAR1 is a transmembrane protein with neuroprotective, neurotrophic, and immunomodulatory properties found in high concentrations in limbic areas of the human brain and in immune cells (Ishikawa et al., 2007; Fujimoto et al., 2012; Frecska et al., 2013; Szabo et al., 2014). SIGMAR1 can be membrane-bound at the mitochondria-associated endoplasmic reticulum (ER) membrane, where it acts as a molecular chaperone, or translocate to the nuclear envelope, the cytosol and the plasma membrane. (Hayashi and Su, 2007; Tsai et al., 2015). At the nuclear envelope, SIGMAR1 recruits chromatin-remodelling molecules to control gene expression (Tsai et al., 2015). Aside from its effects at the ER and nuclear level, SIGMAR1 also plays an important role at the synaptic level both via forming heteroreceptor complexes with G-protein coupled receptors (GPCRs) and via directly interacting with voltage-gated ion channels, therefore controlling the reorganisation of several homo and heteroreceptor complexes and modulating neurotransmission. (Kourrich et al., 2013; Balasuriya et al., 2014; Beggiato et al., 2017; Feltmann et al., 2018; Ortiz-Renteria et al., 2018) Dysregulation of SIGMAR1 function is implicated in neuropsychiatric and neurodegenerative disorders, drug addiction, cancer, cardiovascular diseases, immune-related pathologies, stroke and neuropathic pain [Reviewed in (Tsai et al., 2009; Frecska et al., 2016)].

One peculiarity of the human *SIGMAR1* gene is that, unlike any other, it only shares 30.3% homology with any other mammalian protein, while sharing 66.7% identity with the enzyme sterol isomerase found in fungi, which is involved in the biosynthesis of ergosterol (Hanner et al., 1996; Weete et al., 2010). Ergosterol is a compound found in the cell membrane of fungi and protozoa first identified in the fungus *Claviceps Purpurea* (Weete et al., 2010). Interestingly, this fungus produces ergot alkaloids amongst which lysergic acid, a precursor of the synthetic LSD (Miedaner and Geiger, 2015). Some authors have suggested that, unlike the traditional view that 5HT receptors mediate the psychedelic effects of tryptamines, SIGMAR1 might also be involved in those effects (Fontanilla et al., 2009). Although such discussion is beyond the scope of this work and shall be argued elsewhere, the author believes that SIGMAR1 might represent the real gateway to psychedelic states. Further studies should investigate this possibility.

### Sigma 1 Receptor Activation Is Anti-amnesic

Aside from its neuroprotective and immunomodulatory properties, SIGMAR1 activation has been shown to reverse experimental-induced amnesia in rodents, possibly via enhancement of the cholinergic and *N*-methyl-D-aspartate- (NMDA) glutamatergic neurotransmitter systems (Earley et al., 1991; Maurice et al., 1998; Antonini et al., 2009). Interestingly, peak densities of SIGMAR1 are found in brain areas relevant to traumatic memory formation, retrieval and updating, such as the amygdala and the hippocampal formation, suggesting that Ayahuasca-induced SIGMAR1 activation in such brain areas could be involved in the reported retrieval and updating of traumatic memories (Mash and Zabetian, 1992). Supporting this notion, the parahippocampal gyrus, one of the brain areas hyperactivated by Ayahuasca ingestion, is involved in the modulation of memory retrieval (Woodcock et al., 2015).

Accordingly, SIGMAR1, D2R and 5HT2AR are enriched in the amygdala, while SIGMAR1-D2R and D2R-5HT2AR have been shown to interact to form heteroreceptor complexes at the post-junctional membrane of synapses. (Beggiato et al., 2017; Feltmann et al., 2018) The term “junctional neurotransmission” identifies a type of neurotransmission which is “non-synaptic” and refers to the neurotransmission at neuro-non-neural effectors, that is the connexion between neuronal and non-neuronal cells (such as smooth muscle cells). Such non-synaptic neurotransmission is achieved by means of GPCRs metabotropic receptors signalling, and produces a slower (second to minutes) response compared to synaptic neurotransmission. (Goyal and Chaudhury, 2013) Given that (a) SIGMAR1 plays an important role in junctional neurotransmission via forming heteroreceptor complexes with other metabotropic receptors such as 5HT2AR and D2R and that (b) DMT has high affinity for SIGMAR1 and 5HT2AR, and the latter forms heteroreceptor complexes with both SIGMAR1 and D2R, and that (c) D2R-5HT2AR oligomerization enhances D2R promoter recognition and signalling, it could be plausible that this mechanisms at the post-junctional synapse might enhance the effects of DMT on dopaminergic neurotransmission in the amygdala, a crucial event in memory retrieval and reconsolidation. (Borroto-Escuela et al., 2010, 2014, 2017; Lukasiewicz et al., 2010; Albizu et al., 2011)

Therefore, it seems possible that the elicited patterns of brain activation arising from Ayahuasca ingestion, accompanied by the DMT-induced SIGMAR1 activation leading to gene expression regulation, and by the formation of heteroreceptor complexes to boost dopaminergic neurotransmission, might mediate the retrieval of repressed traumatic memories. This kind of retrieval process forms an essential step in the re-elaboration and re-contextualization of such memories. Interestingly, MAOI activity has also been shown to be beneficial in pre-clinical models of amnesia, and Ayahuasca contains MAOIs (Botwinick and Quartermain, 1974).

### Sigma 1 Receptor Activation Modulates Epigenetic Processes

Recently, SIGMAR1 has been shown to modulate epigenetic processes. In fact, cocaine-induced SIGMAR1 activation triggers SIGMAR1 translocation to the nuclear envelope, where it interacts with proteins which regulate gene expression by affecting chromatin compaction (Tsai et al., 2015). Specifically, SIGMAR1 was shown to create a dose-dependent interaction between emerin and histone deacetylase (HDAC) 1, HDAC2 and HDAC3 and to therefore affect chromatin compaction and gene expression (Demmerle et al., 2012; Tsai et al., 2015). Therefore, for the first time, a study has described an involvement of SIGMAR1 on the epigenetic regulation of gene expression. This is in line with the hypothesis here presented, since reconsolidation and fear extinction of traumatic memories both seem to require the involvement of epigenetic mechanisms (Graff et al., 2014; Kwapis and Wood, 2014).

### Sigma 1 Receptor Activation Disrupts the Reconsolidation of Fear Memories

Aside from promoting anti-amnesic and regulating epigenetic intracellular pathways, activated SIGMAR1 modulates the cannabinoid receptor 1 (CB1)/NMDA receptor interaction to prevent NMDA receptor dysfunction (Sanchez-Blazquez et al., 2014). In support of the hypothesis here presented, CB1 receptors are enriched in the basolateral amygdala (a region involved in conditioned fear), and pharmacological agonists that activate CB1 or NMDA receptors during traumatic memory retrieval disrupt the reconsolidation of fear memories, preventing subsequent fear expression (McDonald and Mascagni, 2001; Lee et al., 2017). This mechanism could be involved in the extinction of fear memory and healing of traumatic memories reported by Ayahuasca users (Nielson and Megler, 2014).

## Memory, Ptsd and Traumatic Memories

Memory formation is the ensemble of highly dynamic processes that permit specific aspects of an event to be stored in the brain (Nadel et al., 2012). The mechanisms involved in memory formation, retrieval and reconsolidation have long been investigated. However, because of the highly complex nature of such processes, and because of the difficulties in studying them, the exact nature of these mechanisms are still debated. It is, however, accepted that mnemonic processes (i.e., memory consolidation, retrieval and reconsolidation) require the involvement of cellular and molecular mechanisms, such as synaptic plasticity and the transcriptional modulation of specific sets of genes in relevant neuronal subpopulations, which are likely to be mediated by epigenetic modifications (Jarome and Lubin, 2014).

Several hypotheses have been formulated trying to describe the elusive mechanisms of memory formation, retrieval and reconsolidation. Although diverging in some aspects, these hypotheses are not necessarily mutually exclusive, and they could hold the key to explain slightly different mechanisms of the same paradigm. The “synaptic plasticity” hypothesis suggests that activity-dependent plasticity is achieved at appropriate synapses and is necessary and sufficient for storage, retrieval and reconsolidation of the memory engram. (Martin et al., 2000; Takeuchi et al., 2014) The “memory indexing theory” or “hippocampal hypothesis of memory,” suggests that the pattern of neocortical and other brain areas activated by an event are initially indexed (i.e., “photographed”) in the hippocampus only to be subsequently stored in other brain regions, such as the neocortex. (Teyler and DiScenna, 1985) The “consolidation hypothesis” disputes that new memories consolidate slowly over time unless new information is learnt shortly after the initial learning. (McGaugh, 2000) The “cholinergic hypothesis” proposes that cholinergic neurons are central player in the formation and storage of memory, and that cholinergic dysfunction is involved in memory and cognitive deficits. (Bartus et al., 1982; Contestabile, 2011) The “vasopressin hypothesis” argues that vasopressin is the fundamental peptide that enhances memory given that vasopressin delayed memory extinction. (Strupp and Levitsky, 1985) The “fragmentation hypothesis” holds that a specific memory is stored as fragments of the specific perceived situation. It has been suggested that this mechanisms might be relevant to PTSD via the phenomenon of “dissociative encoding”, the insufficient encoding of the trauma memory following peritraumatic dissociation which prevents future re-elaboration of the traumatic memory (Bedard-Gilligan and Zoellner, 2012).

Nonetheless, some authors have suggested that long-term memory might be mediated by the allosteric reorganisation of populations of homo- and heteroreceptor complexes in the post-junctional membranes, which in turn affect the pre-junctional receptor complexes to facilitate the new pattern of transmitter release to be learned. (Borroto-Escuela et al., 2015, 2017; Fuxe and Borroto-Escuela, 2016b) Specifically, the transformation of sub-regions of heteroreceptor complexes into transcription factors, upon formation of specific adapter proteins, can consolidate the heteroreceptor complexes into long-term units. Those influence gene expression via altered promoter recognition, signalling and trafficking as well as via the formation of novel allosteric sites, which can lead to changes in promoter function and pharmacology. (Fuxe and Borroto-Escuela, 2016a; Borroto-Escuela et al., 2017) Hence, given that SIGMAR1 controls the reorganisational pattern of several homo- and heteroreceptor complexes at the synapses, it cannot be excluded that these mechanisms might be involved in the reported retrieval and healing of traumatic memories following DMT-mediated SIGMAR1 activation. This might result in a “new post-junctional transmission learning” mediated by a long term modulation of the neuronal networks in which the memory is encoded. Further studies are warranted to explore this possibility.

### Traumatic Memories and Brain Activity in PTSD

When a traumatic event is experienced, the extent of circulating glucocorticoids and adrenalin seem to determine the formation fate of a memory of the event. For example, when the stressor is particularly intense and the levels of stress hormones (such as glucocorticoids) become particularly elevated, formation of the memory can be impaired [Reviewed by (Schwabe et al., 2010)]. Following such events, deficits in declarative memory (the difficulty of recalling the traumatic event) can be experienced (Samuelson, 2011). Exposure to highly traumatic situations can lead to the development of PTSD, a disorder characterised by intrusive thoughts, repression of trauma memory, flashbacks, nightmares, hyperarousal, startle response, and changes in memory and concentration (Bremner, 2006).

Individuals diagnosed with PTSD display changes in brain function and structure, such as alterations of the hippocampus, amygdala and medial prefrontal cortex (including anterior cingulate cortex). Crucially, Ayahuasca ingestion modulates activity of these brain areas (Bremner et al., 1997; Lanius et al., 2001; Bremner, 2006; Riba et al., 2006; Palhano-Fontes et al., 2015). Human studies suggest that PTSD patients present disorder-specific epigenetic regulation of genes involved in pathways relevant to this disorder (Zannas et al., 2015). Given that SIGMAR1 activation is involved in chromatin remodelling and epigenetics regulation of gene expression, it cannot be excluded that DMT-mediated SIGMAR1 activation might affect aberrant gene expression and/or epigenetic signatures in PTSD (Tsai et al., 2015; Zannas et al., 2015).

### Dissociative Amnesia and Brain Activity Changes

A subset of individuals diagnosed with PTSD experience dissociative amnesia, a condition characterised by impaired retrograde memory functioning and loss (or repression) of autobiographic traumatic memory, which is not related to structural brain damage or other cognitive impairments (Brand et al., 2009; Staniloiu and Markowitsch, 2014).

In one imaging study, dissociative amnesia patients displayed hypometabolic functioning of the right inferolateral prefrontal cortex and left supramarginal gyrus (Brand et al., 2009). Another study suggested that dissociative amnesia patients present increased activity in the prefrontal cortex and decreased activity in the hippocampus during a memory retrieval task (Kikuchi et al., 2010). Since Ayahuasca increases brain activity in the parahippocampal region, a brain area involved in memory retrieval, and the hippocampus is underactive during memory retrieval tasks in dissociative amnesia patients, it seems plausible that Ayahuasca might be beneficial in the process of memory recovery in dissociative amnesia patients (Riba et al., 2006; Kikuchi et al., 2010). Interestingly, after treatment for dissociative amnesia, this aberrant pattern of brain activity is reversed (Kikuchi et al., 2010).

### Traumatic Memory Recall in PTSD

During traumatic memory recall in PTSD patients, abnormal brain activity changes can be observed, such as decreased activation of the hippocampus, parietal cortex and decreased activation of the inferior frontal gyrus (the latter is upregulated by Ayahuasca). Moreover, traumatic memory recall hyperactivates the amygdala and posterior cingulate cortex (the latter is hypoactivated following Ayahuasca ingestion) (Bremner, 2006; Palhano-Fontes et al., 2015). Recall and reactivation of a traumatic memory create a plastic window in which the memory becomes labile and has the potential to be updated via epigenetic regulation of neuroplasticity-related gene expression, which can lead to an attenuation (or extinction) of the fear response associated with the memory itself (Nader et al., 2000; Graff et al., 2014). This timeframe (considered to last around 6 h) offers healing potential in PTSD, dissociative amnesia and in any individual that has been exposed to traumatic events (Schiller et al., 2010).

### Memory Reconsolidation and Fear Extinction

The notion that memories are a stable and unchangeable entity has long been disproven. It is now accepted that when memories are retrieved (reactivation of the trace memory), they enter a labile (deconsolidation) state with potential for the memory to be updated (reconsolidation), so that when the memory will be recalled in future, the “new” version of the memory is recalled (Alberini and Ledoux, 2013). However, debate still exists as to whether the new trace memory replaces or co-exists with the old one. Either way, the potential to update memory presents considerable therapeutic implications (Jarome and Lubin, 2014).

The term fear extinction refers to the loss of the fear response associated with the recall of a traumatic memory, and it is an important step in the healing of traumatic memories, such as those experienced by PTSD patients (Myers and Davis, 2007). BDNF-mediated neurogenic processes seem to be essential for this process. (Radiske et al., 2015) In fact, the extinction of the fear responses associated with traumatic memories is mediated by BDNF signalling, and pharmacological BDNF activation after fear extinction hinders the re-emergence of fear (Radiske et al., 2015). Since Ayahuasca increases BDNF signalling, and BDNF mediates fear extinction, it seems plausible that this effect might be involved in the reported processing and amelioration of traumatic memories and in the extinction of the fear response associated with traumatic memories following Ayahuasca ingestion (Fortunato et al., 2009; Morales-Garcia et al., 2017).

### Involvement of Epigenetic Mechanisms in Memory Formation

Epigenetic modifications refer to changes in chromatin compaction which enhance or represses gene transcription and that are not mediated by changes in the underlying DNA sequence (Goldberg et al., 2007). A central role has been suggested for epigenetic regulation in memory processes, since gene transcription seems to be a critical modulator of the processes underlying memory acquisition, retrieval and updating [Reviewed by (Jarome and Lubin, 2014)]. Gene expression shifts result in synaptic functional and structural changes, which lead to changes in synaptic efficacy, thought to underlie the storage and plasticity of memories (Kandel, 2004; Mayford et al., 2012).

### Involvement of Epigenetic Mechanisms in Memory Reconsolidation

The term reconsolidation refers to the updating of an existing memory following retrieval, which results in the updated version of the memory being retrieved in subsequent recall (Alberini and Ledoux, 2013). Relatively little is known about the molecular basis of reconsolidation processes, which are thought to be regulated by intracellular pathways upstream of gene transcription, such as protein kinase A, extracellular signal-regulated kinase/mitogen-activated protein kinase and cAMP responsive element binding protein [Reviewed by (Jarome and Lubin, 2014)].

The nuclear factor kappa-B (NFKB) pathway seems to be critically involved in the epigenetic underpinnings of memory reconsolidation via increasing global histone H3 phosphorylation and acetylation (Lubin and Sweatt, 2007). Significantly, the DMT analogue 5-methoxy-*N,N*-dimethyltryptamine as well as the synthetic psychedelic 2,5-Dimethoxy-4-iodoamphetamine downregulate this pathway, suggesting that DMT and Ayahuasca might present at least same degree of NFKB pathway modulation (Yu et al., 2008; Dakic et al., 2017). It could therefore be argued that Ayahuasca enhances the process of memory reconsolidation via modulating NFKB-mediated regulation of epigenetic modification at the histone level. In support of this notion, a recent study found that activated SIGMAR1 forms a complex with HDAC 1, 2, and 3 and other chromatin remodelling factors to modulate gene expression (Tsai et al., 2015).

Lastly, NMDA receptor activation seems to be essential for memory destabilisation and reconsolidation and it is thought to occur upstream of the mechanisms modulating the epigenetic regulation of gene expression (Gazarini et al., 2014; Jarome and Lubin, 2014). Since activated SIGMAR1 interacts with this receptor, it could be hypothesised that such interaction is involved in the reported beneficial effects of Ayahuasca on memory updating (Sanchez-Blazquez et al., 2014).

### Involvement of Epigenetic Mechanisms in Fear Extinction

Fear extinction is described as a decline in conditioned fear response following exposure to a *non-reinforced* fearful stimulus (i.e., exposure to a context in which the aversive stimulus associated with that context is not presented) (Myers et al., 2006). Fear extinction is thought to involve the “new learning” of a memory that competes with the traumatic one rather than the updating of an old one. Systemic or localised modulation of HDAC activity (and in particular HDAC1 inhibition, which results in histone acetylation and methylation of target genes) seems to be beneficial to fear extinction learning [Reviewed in (Kwapis and Wood, 2014). Since activated SIGMAR1 interacts with HDAC1 and other HDACs, which are involved in fear extinction, and DMT activates SIGMAR1, is possible that DMT and Ayahuasca might be beneficial in erasing the fear response associated with traumatic memories (Tsai et al., 2015).

Similarly, studies have shown that L-type voltage-gated calcium channels (LVGCCs) are involved in the extinction of conditioned fear (McKinney et al., 2008; Davis and Bauer, 2012; Temme and Murphy, 2017). Interestingly, DMT, harmaline and harmane all modulate LVGCCs (Splettstoesser et al., 2005; Johannessen et al., 2009; Gao et al., 2012). Therefore, it seems plausible that such effect might be involved in the Ayahuasca-induced extinction of conditioned fear memories.

“Stronger” memories seem to be more resistant to reconsolidation, and they can become labile only if the retrieval session is prolonged (Besnard et al., 2012). Moreover, the process of extinction of the fear response coupled to a traumatic memory is possible within a window of 6 h after reactivation, while the memory is labile (Schiller et al., 2010). Therefore, the prolonged biological effects arising from Ayahuasca ingestion (3–6 h) could represent a beneficial timeframe for the retrieval, reconsolidation and/or fear extinction of traumatic memories.

## Hypothesis: Ayahuasca Reconsolidates Traumatic Memories Via Modulating Brain Activity, Neurotransmission and Epigenetics

Given the available evidence from cellular, pre-clinical and clinical studies, supported by ample anecdotal evidence, I hypothesise that Ayahuasca ingestion can be helpful in the processing and healing of traumatic memories. I postulate that such effects might be mediated by a multilevel mechanism involving (a) changes in patterns of brain activity conducive to memory retrieval, memory reconsolidation and fear extinction, (b) activation of receptors which trigger anti-amnesic intracellular signalling pathways conducive to the retrieval of traumatic memories, (c) changes in neurotransmitter systems in discrete brain regions which are beneficial to memory updating and fear extinction, (d) enhancement of synaptic plasticity and neurogenesis, and (e) involvement of epigenetic mechanisms, which update the previous emotional association of the memory via modifying the epigenetic signature and cellular memory of the neurons that physically store the traumatic memory. I suggest that these processes result in the memory being updated, to a less- or even non-traumatic form.

Given that Ayahuasca ingestion (a) hyperactivates brain areas involved in the retrieval of memories (parahippocampal gyrus), regulation of emotional processing and perception of errors (anterior insula), regulation and processing of negative emotional stimuli (IFG) and emotional arousal (amygdala), and (b) boosts dopaminergic neurotransmission, an essential requisite for memory retrieval and reconsolidation, I postulate that Ayahuasca creates a pattern of brain activity which is conducive to the recall and/or re-experiencing of traumatic memories, or memories that have a negative connotation (Phillips et al., 1998; Ullsperger et al., 2010; Etkin et al., 2011; Zhu et al., 2012; Tabei, 2015; Urgesi et al., 2016). This process is assisted by the anti-amnesic effects of SIGMAR1 activation, which are potentially mediated by enhanced cholinergic, NMDA-glutamatergic and dopaminergic neurotransmission and might be involved in the retrieval of repressed memories (Earley et al., 1991; Maurice et al., 1998; Antonini et al., 2009).

When the traumatic memory is recalled, it enters a labile state, which allows for the memory to be updated and reconsolidated and/or for the fear response associated with the memory to be erased (fear extinction). In line with this notion, Ayahuasca hyperactivates the subcallosal gyrus, which is involved in the rehearsing and processing of “sad” autobiographic memories, while hyperactivating and boosting dopaminergic neurotransmission in the amygdala; these processes are involved in fear extinction (Mayberg et al., 1999; Riba et al., 2006; Myers and Davis, 2007; Palhano-Fontes et al., 2015). These effects suggest the instauration of a pattern of brain activity which is conducive to the processing of traumatic memories and to the extinction of the conditioned fear response associated with such memories (Mayberg et al., 1999; Myers and Davis, 2007).

SIGMAR1 has been shown to modulate the CB1/NMDA receptor interaction (Sanchez-Blazquez et al., 2014). Since CB1 receptors are enriched in the amygdala (one of the brain areas hyperactivated by Ayahuasca), and given that CB1 or NMDA agonists prevent fear expression during traumatic memory retrieval, this supports a role for Ayahuasca in the updating and reconsolidation of traumatic memories by modulating the fear responses associated with the traumatic memory (McDonald and Mascagni, 2001; Lee et al., 2017). The hypothesis presented in this paper is also supported by the fact that each of the main alkaloids present in Ayahuasca modulate LVGCCs, which are involved in the extinction of conditioned fear (Splettstoesser et al., 2005; McKinney et al., 2008; Johannessen et al., 2009; Davis and Bauer, 2012; Gao et al., 2012; Temme and Murphy, 2017).

Moreover, given the neurotrophic and neurogenic effects of the alkaloids present in Ayahuasca, it cannot be excluded that these effects too could be involved in the processes of memory reconsolidation and/or fear extinction, since those mnemonic processes require increased synaptic plasticity (Fortunato et al., 2009; Radiske et al., 2015; Morales-Garcia et al., 2017). Furthermore, Ayahuasca ingestion increases dopaminergic and decreases GABAergic neurotransmission in the amygdala (de Castro-Neto et al., 2013). In line with the hypothesis here presented, the former is involved in fear extinction learning, while negative regulation of the latter is conducive to anti-amnesic effects (Barad et al., 2006; Rau et al., 2009). These lines of evidence support a role for Ayahuasca in the retrieval of traumatic, repressed memories and in the extinction of the fear associated with such memories in PTSD and dissociative amnesia (Barad et al., 2006; de Castro-Neto et al., 2013).

Finally, SIGMAR1 interacts with the epigenetic modulators HDAC1, 2 and 3, and these proteins are involved in transcriptional regulation and chromatin remodelling, representing crucial modulators of memory updating and reconsolidation in the amygdala (Maddox and Schafe, 2011). Hence, I hypothesise that DMT-mediated SIGMAR1 activation, via the recruitment of HDAC and other chromatin remodelling molecules, results in the updating of the traumatic memory. This occurs by means of epigenetic modifications in the neurons that previously stored the traumatic memory; these modifications delete the transcriptional “instructions” to trigger the fear response that is associated with the memory updating the memory to become a non-traumatic one.

To the best of the author’s knowledge, no formal study has investigated the potential of Ayahuasca or its alkaloids in isolation in the updating of traumatic memories and in fear extinction processes. However, one study has investigated the potential of psilocybin in the extinction of fear conditioning. In that study, mice receiving low doses (but not high doses) of psilocybin, exhibited a facilitated extinction of the fear response in a paradigm for hippocampal-dependent trace conditioning paradigm (Catlow et al., 2013). A mechanistic explanation for such differences was not investigated in that study. The authors suggested that, since psilocybin increases dopaminergic neurotransmission, and since drugs that increase dopamine availability are beneficial to fear extinction, psilocybin might be beneficial to fear extinction via enhancing dopaminergic neurotransmission (Aghajanian and Marek, 1999; Vollenweider et al., 1999; Vazquez-Borsetti et al., 2009; Catlow et al., 2013). A similar study investigating the effects of 3,4-methylenedioxy-methamphetamine (MDMA) on fear extinction in rodents found that MDMA enhanced fear extinction when administered before fear extinction learning while increasing BDNF signalling in the amygdala following extinction learning (Young et al., 2015).

## Translational Implications of Ayahuasca-Mediated Healing of Cellular Memory

Cellular memory is the maintenance of gene expression and silencing patterns in a cell through the processes of DNA replication and packaging, which almost never involves changes in DNA sequence (Turner, 2002; Henikoff and Greally, 2016). This type of intrinsic DNA-bound memory is crucial during development and later in life to rule out cellular transformation (Jacobs and van Lohuizen, 2002). Given the influence of activated SIGMAR1 on epigenetics processes and chromatin compaction, it seems possible that DMT and Ayahuasca might modulate cellular memory (Tsai et al., 2015).

Cellular memory is mediated by proteins and short non-coding RNAs which, following DNA replication, ensure the perpetuation of repressed and active transcriptional states in the offspring. Examples are proteins belonging to the polycomb and trithorax families, and microRNAs, which are involved in cellular memory, reprogramming and differentiation by controlling the expression of hundreds of target genes (Steffen and Ringrose, 2014; Stuwe et al., 2014; Hwang et al., 2017).

Cellular memory seems to be dysregulated in many conditions, including but not limited to PTSD, cancer, epilepsy, neurodegenerative and gastrointestinal disorders and gut microbiome-mediated diseases (Jacobs and van Lohuizen, 2002; Paul et al., 2015; Zannas et al., 2015; Hwang et al., 2017; Kiese et al., 2017). Drugs that modulate cellular memory have the potential to reverse pathological cellular phenotypes mediated by dysregulated cellular memory. Examples include molecules able to change the fate of cells with dysregulated cellular memory in cancer and those able to restore neuron-mediated synaptogenesis and learning in neurodegeneration (Henikoff and Greally, 2016; Hwang et al., 2017).

Therefore, given that SIGMAR1 interacts with the epigenetic machinery, and given that the alkaloids present in Ayahuasca have neurogenic, neuroplastic, neuroprotective and immunomodulatory properties, it seems plausible that these compounds might be useful in diseases such as those of neurodegenerative and transformative nature (i.e., Alzheimer’s disease and cancer) (Jacobs and van Lohuizen, 2002; Fortunato et al., 2009; Tsai et al., 2015; Hwang et al., 2017; Morales-Garcia et al., 2017). This represents a tentative suggestion and further studies should investigate this possibility.

## Future Directions

Further studies are required to determine if the hypothesis here presented is indeed confirmed by experimental evidence. If the theory here postulated proves to be true, Ayahuasca could represent the first pharmacological therapy which targets traumatic memories in PTSD and dissociative amnesia.

Double-blind, placebo-controlled studies are warranted to determine the efficacy of Ayahuasca treatment in PTSD and dissociative amnesia. Brain activity in PTSD patients could be assessed via imaging techniques before and during Ayahuasca ingestion, to assess potential regional changes in activity which could indicate improvements of PTSD symptoms. PTSD and dissociative amnesia patients could be asked to rehearse traumatic memory during Ayahuasca ingestion to determine if this could indeed represent a helpful approach in relieving the traumatic connotation of those memories. Moreover, follow up studies could investigate if the abnormal patterns of brain activation in PTSD and dissociative amnesia patients can be altered by Ayahuasca ingestion and if such alterations are stable over time (Lanius et al., 2001; Brand et al., 2009). Particular care should be taken in these settings, because the retrieval of repressed traumatic memories could inadvertently result in re-traumatization (Nielson and Megler, 2014). Therefore, psychological support should be available before, during and after such trials, to ensure adequate care for the well-being of the participants. Nevertheless, because fear extinction is generally considered to be susceptible to change rather than permanent, it is important to follow-up PTSD patients that undergo Ayahuasca therapy for the healing of traumatic memories; there exists the possibility that the fear memory might be *reinstated*, *renewed*, or *spontaneously recovered* (Myers and Davis, 2007).

Pre-clinical studies could investigate the potential of Ayahuasca treatment through paradigms of fear extinction. Such paradigms include contextual fear conditioning and cued fear conditioning. Contextual fear conditioning involves (a) placing the animal in a novel environment, (b) presenting them with an aversive stimulus, (c) removing the animal, (d) subsequently returning the animal to the same environment and quantifying “freezing” behaviour without presenting the aversive stimulus. Cued fear conditioning paradigms are similar to contextual ones with the difference that the aversive stimulus is preceded by a contextual cue (i.e., a sound or a light). When the animals are returned to the testing environment, the cue is presented but the aversive stimulus is not and freezing behaviour is quantified. The freezing behaviour derives from the fact that the animal has learned that the specific environment is unpleasant and being placed in such environment will likely result in the aversive stimulus being re-presented (Curzon et al., 2009). If Ayahuasca-treated animals present decreased freezing behaviour in those paradigms, this might suggest that indeed Ayahuasca is beneficial in the extinction of fear conditioning, as it was previously demonstrated with 3,4-methylenedioxymethamphetamine and low-dose psilocybin (Catlow et al., 2013; Young et al., 2015).

To the best of the author’s knowledge, no study has investigated the effects of Ayahuasca or other psychedelic compounds on processes involving epigenetic regulation of gene expression. However, studies investigating gene expression changes stemming from LSD and proteomic changes induced by the DMT analogue 5-MeO-DMT have been reported (Nichols and Sanders-Bush, 2002; Nichols et al., 2003; Dakic et al., 2017). An early investigation into long-term Ayahuasca drinkers suggested that long-term Ayahuasca consumption increases 5HT binding sites in platelets (Callaway et al., 1994). Although this study did not suggest an involvement of epigenetic processes in the changes observed, it cannot be excluded that the differences observed might be mediated by changes in epigenetic regulation of the 5HT receptor gene or neighbouring regulatory DNA regions.

Further studies should investigate the possibility that Ayahuasca affects epigenetic processes and epigenetic markers thereof (such as histone acetylation, methylation and phosphorylation and DNA methylation). Such studies could involve either epigenome-wide interrogation or analyses at specific DNA (or histone) sites of interest in specific pathologies, such as PTSD, depression, and autoimmune disorders.

Finally, in order to investigate if Ayahuasca has effects on cellular memory mechanisms, studies could be performed analysing the expression levels and epigenetic signatures of proteins involved in cellular memory regulation, such as those belonging to the polycomb and trithorax families, which, respectively, sustain repressed and active transcriptional states of hundreds of genes (Steffen and Ringrose, 2014). If Ayahuasca indeed affects the transcription, translation, post-translational modification or epigenetic regulation of those proteins, it could mean that Ayahuasca has an effect on cellular memory processes. If this proves to be true, it could be that the beneficial effects observed and reported in several diseases and conditions following Ayahuasca ingestion might be mediated by its SIGMAR1-mediated effects on those master regulatory mechanisms (Frecska et al., 2016; Henikoff and Greally, 2016).

## Conclusion

In this work a mechanism that might explain the healing effects of Ayahuasca ingestion on traumatic memories has been hypothesised. I have highlighted the effects of Ayahuasca on the modulation of brain activity, neurotransmission, and neurogenesis, which are consistent with the reported effects of Ayahuasca on the retrieval of traumatic memories. Moreover, I have explored the effects of DMT-mediated SIGMAR1 activation on the modulation of anti-amnesic pathways and on the regulation of epigenetic processes, which might be involved in the healing of traumatic memories via memory reconsolidation and/or fear extinction. Moreover, given the effects of SIGMAR1 on epigenetic processes, the author suggests that Ayahuasca might be useful in the treatment of disorders in which the cellular memory is dysregulated, such as cancer and neurodegenerative and autoimmune diseases. Further efficacy studies should aim at optimising therapeutic Ayahuasca doses while investigating the hypothesis here presented in randomised controlled trials with PTSD and dissociative amnesia patients and/or in pre-clinical models of PTSD, memory reconsolidation and fear extinction. Clinical trials should be coupled to preliminary psychiatric assessments, ongoing psychological treatment and integration to avoid negative psychological outcomes, such as fear renewal, fear reinstatement or psychosis. If the hypothesis here presented proves to be true, it might help guide evidence-based informed policy decisions. Such decisions could help alter the legal status of Ayahuasca and lead toward the utilisation of Ayahuasca in psychiatry and other fields of medicine.

## Author Contributions

The author confirms being the sole contributor of this work and approved it for publication.

## Conflict of Interest Statement

The author declares that the research was conducted in the absence of any commercial or financial relationships that could be construed as a potential conflict of interest.

## Abbreviations

5HT: Serotonin
BDNF: Brain-derived neurotrophic factor
CB1: Cannabinoid receptor 1
DMN: Default mode network
DMT: *N,N*-Dimethyltryptamine
GABA: Gamma-aminobutyric acid
HDAC: Histone deacetylase
IFG: Inferior frontal gyrus
LVGCC: L-type voltage-gated calcium channels
MAOI: Monoamine oxidase inhibitor
MDMA: 3,4-methylenedioxy-methamphetamine
NFKB: Nuclear factor kappa-B
NMDA: *N*-methyl-D-aspartate
PTSD: Post-traumatic stress disorder
SIGMAR1: Sigma 1 receptor
